# Combining effect of camellia oil and squalene on hyperlipidemia-induced reproductive damage in male rats

**DOI:** 10.3389/fnut.2022.1053315

**Published:** 2022-11-18

**Authors:** Qi Xu, Minhui Luo, Gengjinsheng Cheng, Qi Zhong, Yixing Guo, Jianghong Luo

**Affiliations:** ^1^School of Public Health and Health Management, Gannan Medical University, Ganzhou, China; ^2^Department of Quality Control, Ganzhou People’s Hospital, Ganzhou, China; ^3^College of Pharmacy, Gannan Medical University, Ganzhou, China; ^4^Key Laboratory of Prevention and Treatment of Cardiovascular and Cerebrovascular Diseases, Ministry of Education, Gannan Medical University, Ganzhou, China

**Keywords:** camellia oil, squalene, hyperlipidemia model, improve erectile and sexual function, combining effect

## Abstract

**Introduction:**

Camellia oil (CO), a common edible oil in China, contains a variety of active ingredients. In this study, we explored the combining effect and optimal feeding time of CO and squalene on hyperlipemia-induced reproductive damage rats and probably provided supportive data for use of CO for health benefits.

**Methods:**

We established the hyperlipidaemia-induced reproductive damage model, and then the successfully modeled rats were randomly classified into four groups including a model control (MC) group, a camellia oil (CO) group, a camellia oil + squalene (COS) group, and a sildenafil (SN) group, which were feeding with different subjects during days 30 and 60. The normal (NC) group was fed under the same conditions.

**Results:**

Our results showed that compared with the MC group, the CO, COS, and SN groups could significantly decline the serum TG, TC and LDL-C levels, increase the serum testosterone levels, the sperm counts in epididymidis and organ coefficients of penises, and no pathological change in penis and testis at days 30 and 60. Compared with the pure CO, the mixture of CO and squalene could significantly enhance the effect of decreasing the concentrations of TG, TC, and LDL-C and increasing the serum testosterone level and sperm count of epididymal tail, and the results of day 30 were better than those of day 60.

**Discussion:**

CO and squalene have a combining effect on lowering blood lipid, improving the level of testosterone and the number of epididymal tail sperm, and promoting the recovery of erectile and sexual function on hyperlipidemia-induced reproductive damage rats on day 30.

## Introduction

Hyperlipidemia, a common metabolic syndrome (1), the typical symptoms include the abnormal elevation of any or all lipids or lipoproteins in the blood (2). Hyperlipidemia is a critical damage-inducing factor for cardiovascular disease and frequently brings about many complications, such as cardiac damage (3), sexual dysfunction (4), cognitive impairment (5), inflammation, and insulin resistance (6). Strong associations are observed between hyperlipidemia and sexual dysfunction especially erectile dysfunction (ED). Experimental studies showed that the reduction in arterial blood flow induced by hyperlipidemia can directly affect the functions of cortical center, pituitary–testis axis, and corpus cavernosum penis (7) and inhibit the production of testosterone (8), which cause a decline in sexual function. Nowadays, many synthetic pharmaceuticals, like sildenafil (SN), are widely used for the management of ED. However, their long-term use always causes many serious side effects, including dizziness, headaches, heartburn, indigestion, stuffy nose, and vasodilatation (9). Given the potential risks and side effects associated with current treatments, it is important to explore more efficient and safe candidates.

Meanwhile, many promising sources can be used to regulate and manage ED (10). Some studies showed that black tea brew possessed marked aphrodisiac activity in terms of prolongation of latency of ejaculation shortening of mount- and intromission latencies and elevation of serum testosterone level (11). Moringa oleifera Lam. Leaf Tea could enhance sexual function and the male reproductive system (12). Camellia oil (CO), which obtain from the seeds of Camellia oleifera Abel, is a common edible oil in China and contains a variety of active ingredients (13, 14), such as Squalene, which can raise the activity of internal Superoxide Dismutase (SOD) and blood oxygen content (15), stimulate blood circulation (16), and improve sexual function (17). Vitamin E can promote the secretion of sex hormones and maintain the normal function of the Genital organs (18). Camellia Saponin can reduce serum cholesterol and prevent cardiovascular diseases (19, 20). The research group observed in a previous study is that CO can lower blood lipids, promote the secretion of the sex hormone testosterone, and improve sexual function (21). To further enhance the effectiveness of camellia oil, in this research, squalene, an active ingredient of CO, is proposed to be added to feed rats with hyperlipidemia-induced sexual dysfunction as a combining substance and observe the changes in blood lipid and testosterone levels, epididymal sperm count, and testis and corpus cavernosum tissue structures. This study aims to explore the combining effect and optimal feeding time of CO and squalene on hyperlipidemia-induced sexual dysfunction rats and provide supportive data for the health benefits of CO use.

## Materials and methods

### Materials and chemicals

High-purity CO purchased from Jiang Xi Qiyunshan Food Co., Ltd., (Ganzhou, China) contained 78.8/100 g monounsaturated fatty acid, 9.0/100 g linoleic acid, 2.3/100 g linolenic acid, 1.3/100 g stearic acid, 8.6/100 g palmitic acid, 0.0/100 g protein, 0.0/100 g cholesterol, and 0.0/100 g sugar.

Basal and high-fat feeds were provided by Guangdong Medical Laboratory Animal Center (Guangdong, China). High-fat feed (100 g) consisted of 78.8 g basal feed, 10.0 g lard, 10.0 g yolk powder, 1 g cholesterol, and 0.2 g cholate.

Squalene and SN were purchased from Beijing InnoChem Science and Technology Co., Ltd., (Beijing, China). Total cholesterol (TC), triglyceride (TG), and low-density lipoprotein cholesterol (LDL-C) test kits were obtained from Nanjing Jiancheng Bioengineering Institute (Nanjing, China). The testosterone ELISA kit was purchased from Cloud-Clone Corp., Wuhan (Wuhan, China). The hematoxylin–eosin (HE) dye was acquired from Beijing Zhongshan Jinqiao Biotechnology Co., Ltd., (Beijing, China). All other used reagents were analytical grade.

### Animals

Specific pathogen-free male Sprague Dawley rats (weight = 150–180 g) (Certificate number SCXK [xiang] 2019-0004) were purchased from Hunan Slac Jingda Laboratory Animal Co., Ltd., (Hunan, China). Animals were housed in cages in a specific pathogen-free room with controlled temperature (18–26°C), relative humidity (40–60%), and 12/12 h of light–dark periods with *ad libitum* food and water prior to experiments. All procedures were approved by the Animal Experiment Ethics Committee, Gannan Medical University, China.

### Animal experiment design

Rats were fed with a high-fat diet to establish a hyperlipidemia-induced reproductive damage model. Rats were randomly divided into the normal (NC, 12 rats) and model (MC, 48 rats) control groups and supplied with basal and high-fat feeds, respectively, for 4 weeks. Serum TC, TG, LDL-C, and testosterone levels of rats were measured using the corresponding kits in accordance with the manufacturer’s instructions. Compared with the NC group, the MC group showed significant increments in serum TC, TG, and LDL-C levels and prominent reduction in the serum testosterone content.

The successfully modeled rats were randomly classified into four groups (12 mice per group): Converted by recommended intake of oils and fats, squalene, SN of human and “equivalent dose ratio table of human and animal body surface area conversion.” The MC group received 3 mL/kg⋅bw⋅day of 0.9% normal saline. The CO group was treated with 3 mL/kg⋅bw⋅day of CO. The CO + squalene (COS) group was supplemented with 3 mL/kg⋅bw⋅day suspension solution (CO: squalene = 30:1) containing CO and squalene. The SN group was supplemented with 3 mL/kg⋅bw⋅day suspension solution (CO: sildenafil = 3:1) containing CO and sildenafil. Rats in the NC group were orally administered with 3 mL/kg⋅bw⋅day of 0.9% normal saline. During the experimental period, blood was collected at days 30 and 60. Half of the rats in each group were sacrificed at days 30 and 60. The testis, epididymis, and penis of rats were obtained.

### Sexual organ index determination

The obtained testis, epididymis, and penis were rinsed with 0.9% normal saline, blotted dry using filter paper, and weighed. Organ indices were calculated using the equation: weight of organ/weight of rat.

### Serum lipid and testosterone levels measurement

The blood of rats was centrifuged at 3,000 rpm and 4°C for 10 min to collect the serum. TC, TG, LDL-C, and testosterone levels of serum were detected using assay kits as specified by the manufacturer.

### Sperm count metering in epididymidis

The cauda of epididymidis was cut off, placed into 4 mL of 0.9% normal saline, and cut. The cauda of the epididymidis was incubated at 37°C for 20 min to allow running out of sperm. Afterward, the supernatant was obtained through filtration by using 100-mesh strainer. The supernatant (10 μL) was transferred into the sperm count board, and the sperm number was counted under electron microscopy.

### Hematoxylin–eosin staining observation of testis and penis

Parts of testis and penis were fixed in 10% formalin for over 24 h and embedded in paraffin. Then, paraffin sections (5 μm thick) were stained with HE dye and histopathologically observed using electron microscopy.

### Statistical analysis

All data were presented as mean ± standard deviation. Statistical analysis was carried out using the SPSS 23.0 software (SPSS Inc., Chicago, IL, United States). One-way analysis of variance was adopted to compare significant differences among all groups by Tukey’s analysis. Two-tailed *p* < 0.05 was considered statistically significant.

## Results

### Hyperlipidemia-induced reproductive damage in high-fat diet model

**FIGURE 1:**
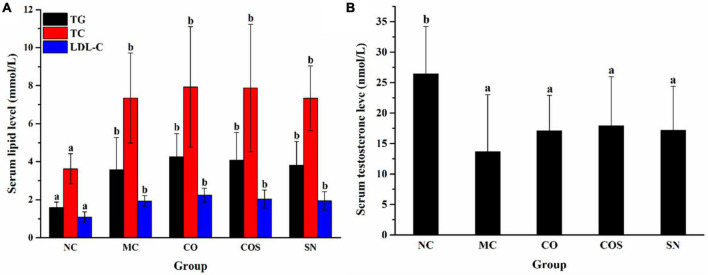
Serum lipid **(A)** and testosterone **(B)** levels of rats at the 30th day in hyperlipidemia-induced reproductive damage modeling. TG, triglyceride; TC, total cholesterol; LDL-C, low-density lipoprotein cholesterol; NC, normal control; MC, model control; CO, Camellia oil; COS, Camellia oil + squalene; SN, sildenafil. Different letters represent significant differences (*p* < 0.05).

The elevation of serum TC, TG, and LDL-C levels is the typical symptom of hyperlipidemia (22). As shown in Figure 1A, the TC, TG, and LDL-C levels of rats in high-fat diet treatment groups, including MC, CO, COS, and SN groups, were significantly elevated compared with those in the NC group. Results indicated that the hyperlipidemia model was successfully established. The serum testosterone levels of rats were determined as illustrated in Figure 1B to evaluate whether hyperlipidemia induced reproductive damage. Compared with the NC group, the hyperlipidemia group had prominently decreased serum testosterone level, and this finding was consistent with those of previous reports (21, 23, 24). This result suggested that hyperlipidemia-induced reproductive damage occurred in the high-fat diet model.

### Combining effect of camellia oil and squalene on hyperlipidemia-induced reproductive damage in male rats

#### Sexual organ indexes of rats

The sexual organ (i.e., testis, epididymis, and penis) indices of rats are shown in Table 1. No significant difference was observed in the testis and epididymis organ indices of rats among NC, MC, CO, COS, and SN groups. Compared with the MC group, the NC, CO, COS, and SN groups had significantly increased penis organ indices at days 30 and 60, suggesting that the CO, COS, and SN groups could improve the relaxation and atrophy of corpus cavernosum penis in rats.

**TABLE 1:** Sexual organ indices of hyperlipidemia-induced reproductive damage rats.

Group	Testis index (g/100 g)		Epididymis index (g/100 g)		Penis index (g/100 g)	
	Day 30	Day 60	Day 30	Day 60	Day 30	Day 60
NC	0.791 ± 0.078^a^	0.762 ± 0.025^a^	0.243 ± 0.022^a^	0.220 ± 0.016^a^	0.082 ± 0.010^a^	0.084 ± 0.018^a^
MC	0.795 ± 0.082^a^	0.750 ± 0.086^a^	0.248 ± 0.023^a^	0.220 ± 0.041^a^	0.069 ± 0.011^b^	0.061 ± 0.013^b^
CO	0.778 ± 0.080^a^	0.727 ± 0.082^a^	0.251 ± 0.012^a^	0.222 ± 0.027^a^	0.080 ± 0.008^a^	0.078 ± 0.012^a^
COS	0.799 ± 0.095^a^	0.748 ± 0.077^a^	0.241 ± 0.020^a^	0.227 ± 0.033^a^	0.081 ± 0.008^a^	0.082 ± 0.010^a^
SN	0.757 ± 0.073^a^	0.733 ± 0.054^a^	0.245 ± 0.021^a^	0.226 ± 0.031^a^	0.085 ± 0.011^a^	0.079 ± 0.011^a^

NC, normal control; MC, model control; CO, Camellia oil; COS, Camellia oil + squalene; SN, sildenafil. Values in the same column or row with different letters represent significant differences (*p* < 0.05).

#### Serum lipid levels of rats

**FIGURE 2:**
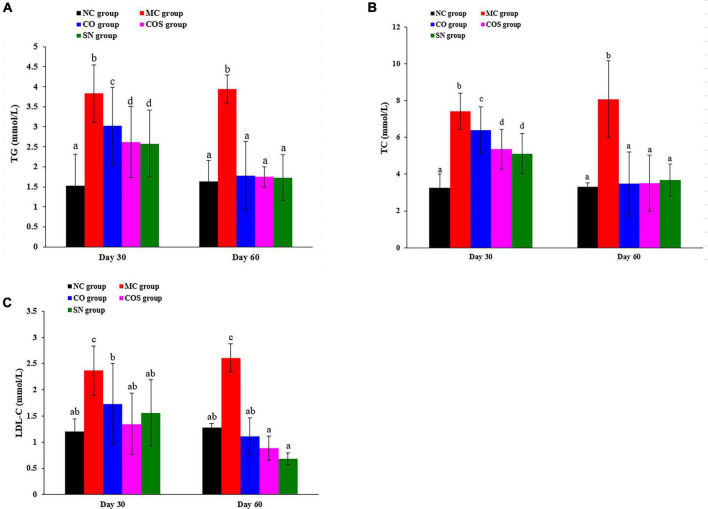
After the end of modeling, serum lipid levels of hyperlipidemia-induced reproductive damage rats at the another 30th and 60th day by feeding test substance: **(A)** TG, **(B)** TC, and **(C)** LDL-C levels. TG, triglyceride; TC, total cholesterol; LDL-C, low-density lipoprotein cholesterol; NC, normal control; MC, model control; CO, Camellia oil; COS, Camellia oil + squalene; SN, sildenafil. All values are expressed as mean ± SD. Different letters represent significant differences (*p* < 0.05).

The serum lipid (i.e., TG, TC, and LDL-C) levels of normal and hyperlipidemia rats are shown in Figure 2. Compared with the NC group, the serum lipid levels of rats were notably increased in the MC group at days 30 and 60. The treatments of test substances (i.e., CO and COS) could reverse this phenomenon. As illustrated in Figures 2A,B), at day 30, the serum TG and TC levels of rats in CO, COS, and SN groups declined compared with those in the MC group. Meanwhile, the serum TG and TC levels of rats in COS group declined compared with those in the CO group, indicating that CO and squalene could significantly enhance the effect of decreasing the serum TG and TC levels of hyperlipidemia rats. At day 60, the serum TG and TC levels of rats in the CO and COS groups notably decreased, while there was no significant difference between CO and COS groups. Figure 2C reveals that the LDL-C levels of rats supplemented with the above test substances (i.e., CO and COS) were markedly lower than those in the MC group at days 30 and 60.

#### Serum testosterone level of rats

**FIGURE 3:**
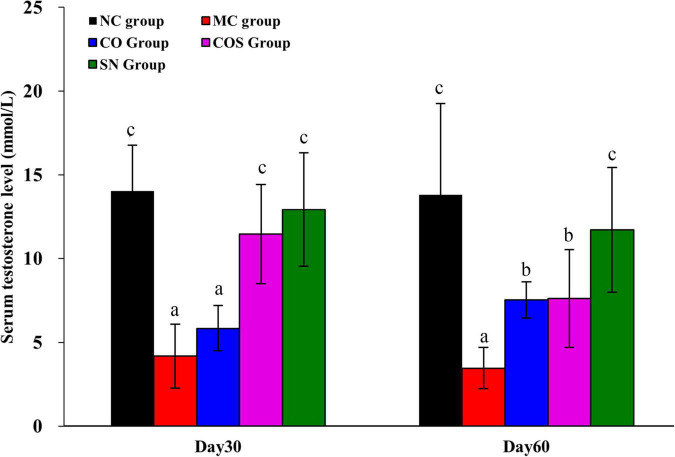
After the end of modeling, serum testosterone levels of hyperlipidemia-induced reproductive damage rats at the another 30th and 60th day by feeding test substance. NC, normal control; MC, model control; CO, Camellia oil; COS, Camellia oil + squalene; SN, sildenafil. All values are expressed as mean ± SD. Different letters represent significant differences (*p* < 0.05).

The serum testosterone levels of rats were detected (Figure 3). Compared with those in the NC group, the serum testosterone levels of rats in the MC group significantly decreased at days 30 and 60. At day 30, the serum testosterone levels of rats in the COS and SN groups dramatically increased, whereas no outstanding increment was observed in the CO group. Results suggested that CO and squalene exerted combining effect against hyperlipidemia-induced reduction in the serum testosterone level of rats. At day 60, rats in the CO, COS, and SN groups showed significant elevation in serum testosterone levels in comparison with those in the MC group. At day 30, the serum testosterone level showed varying degrees of decrease in the COS and SN groups.

#### Sperm count in epididymidis of rats

**FIGURE 4:**
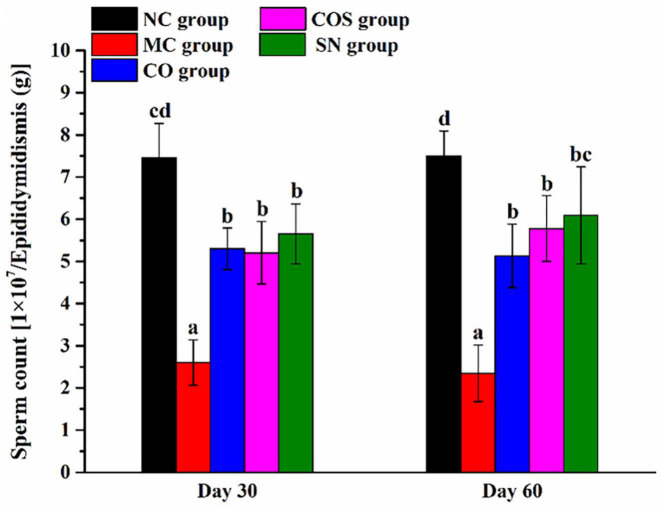
Sperm counts in the epididymidis of hyperlipidemia-induced reproductive damage rats. NC, normal control; MC, model control; CO, Camellia oil; COS, Camellia oil + squalene; SN, sildenafil. All values are expressed as mean ± SD. Different letters represent significant differences (*p* < 0.05).

The sperm counts in the epididymidis of rats are shown in Figure 4. Compared with those in the NC group, the sperm counts in the epididymidis of rats in the MC, CO, COS, and SN groups were prominently reduced. The oral administration of CO, COS, and SN could significantly increase the sperm counts in the epididymidis of rats at days 30 and 60 compared with that of MC. The sperm counts in the epididymidis of rats in all groups at day 60 were not significantly different compared with those at day 30.

#### Histomorphologies of testis and penis

**FIGURE 5:**
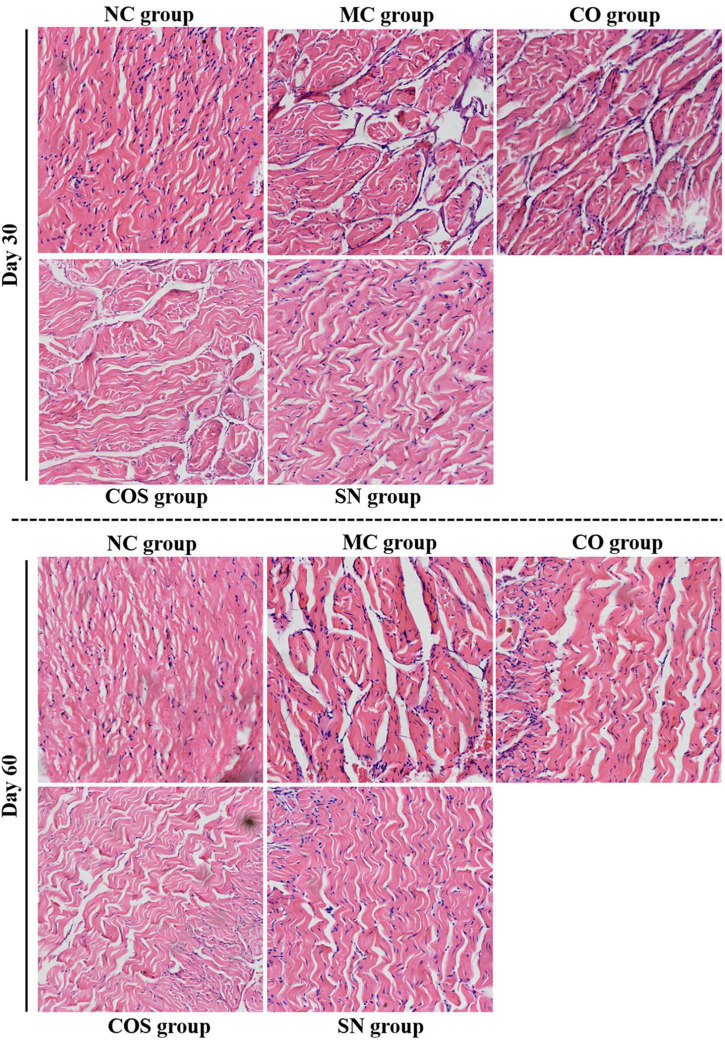
Penis histomorphologies of hyperlipidemia-induced reproductive damage rats. Photomicrographs of penis tissue sections stained with hematoxylin and eosin (H&E) (× 200). NC, normal control; MC, model control; CO, Camellia oil; COS, Camellia oil + squalene; SN, sildenafil.

**FIGURE 6:**
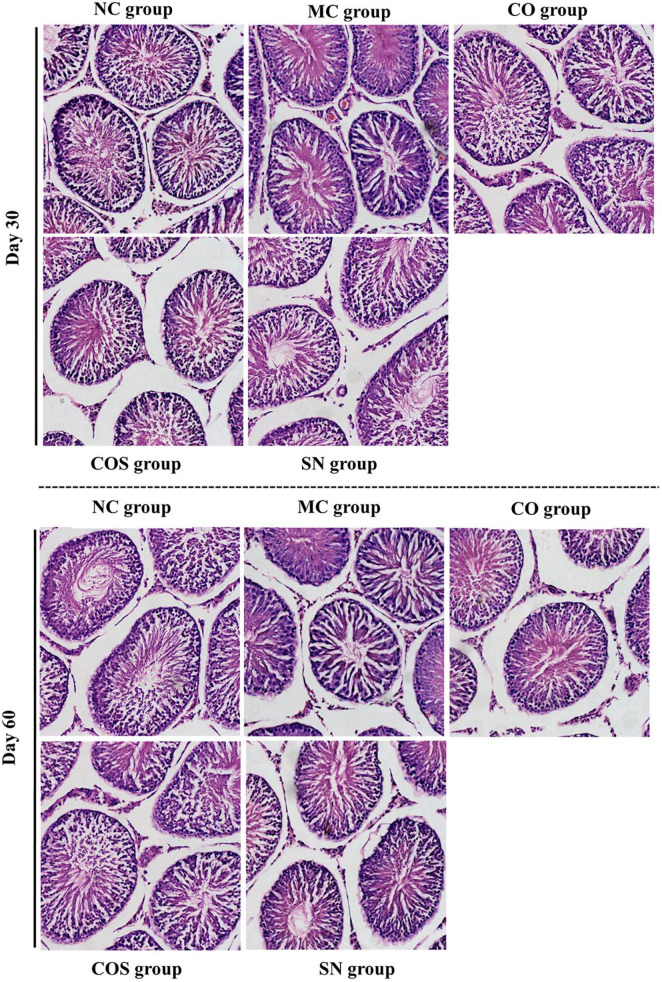
Testis histomorphologies of hyperlipidemia-induced reproductive damage rats. Photomicrographs of testis tissue sections stained with hematoxylin and eosin (H&E) (× 200). NC, normal control; MC, model control; CO, Camellia oil; COS, Camellia oil + squalene; SN, sildenafil.

The histomorphologies of testis and penis are shown in Figures 5, 6. Compared with the NC group, the CO, COS, and SN groups had no pathological change in the corpus cavernosum penis and testes. In the MC group, the number of smooth muscle cells and cavernous sinus decreased significantly in the corpus cavernosum penis, and the smooth muscle of the cavernous body were distributed unevenly and arranged disorderly and loosely. At the same time, all levels of spermatogenic cells in the testis were arranged disorderly, and some spermatogenic cells showed necrosis, apoptosis, and nuclear pyknosis at days 30 and 60.

## Discussion

Hyperlipemia is a prevalent, chronic condition that affects penile hemodynamics (25), Huang et al. found that hyperlipidemia could damage arterial endothelial cell function through cell adhesion molecule-1, cause inadequate blood supply to the penis and reduced blood flow to the Corpus cavernosum penis arteries, which could lead to erectile dysfunction and sexual dysfunction by causing of cavernosal fibrosis (26). At present, researches on tea mainly focus on the effect of tea polyphenols on hyperlipidemia (27) and reproductive system (19, 28, 29), Jiraporn et al. (12) reported that due to Moringa oleifera Lam. Leaf Tea containing rich total phenols, flavonoids, and antioxidants, it could enhance sexual function and the male reproductive system. However, the effect of Camellia oil and its active ingredient squalene on hyperlipidemia-induced sexual dysfunction rats has not been reported.

This study was designed to explore the combining effect and optimal feeding time of CO and squalene on hyperlipidemia-induced sexual dysfunction rats. As expected, the hyperlipidemia rat model was successfully induced by feeding with a high-fat diet for 4 weeks (30). The serum levels of TC, TG, and LDL-C of hyperlipidemic rats orally administered with CO and COS significantly decreased compared with those orally administered with MC at day 60. The results of our study indicated that CO can significantly reduce blood lipids that are rich in monounsaturated fatty, oleic, linoleic, and linolenic acids. This is in agreement with other studies (19, 30–32). Furthermore, we studied the effect of camellia oil on reducing blood lipids at different doses in our previous study (21), including very low (0.75 mL/kg⋅bw⋅day), low (1.5 mL/kg⋅bw⋅day), medium (3 mL/kg⋅bw⋅day) and high (4.5 mL/kg⋅bw⋅day) dose, and found that medium (3 mL/kg⋅bw⋅day) dose was optimal. Therefore, in this present study, we investigated this dose of camellia oil with its active ingredient squalene to observe their combining effect on reducing blood lipids. The results indicated that compared with CO group, COS group significantly reduced the level of blood lipids at day 30, suggesting that CO and squalene could play an enhancing role in combining the effect of reducing blood lipids, and this effect was better than that of pure CO. Studies showed that squalene probably plays a role in hindering the intestinal cholesterol absorption or restraining the activities of key enzymes, such as hepatic acyl-CoA oxidase, fatty acid synthase, and hydroxyl-3-methylglutarylcoenzyme A reductase in endogenous cholesterol biosynthesis (33, 34).

Testosterone, the main male hormone in the body, can promote the development of male reproductive organs and sperm and maintain sexual function (35). Wannasiri et al. (36) and Vigueras-Villaseñor et al. (37) induced hyperlipidemia in male Wistar or Sprague-Dawley rats by feeding with a high-fat diet, found that the testosterone level was decreased in the high-fat diet group. It is proposed that elevated estradiol levels derived from peripheral aromatization of androgen inhibits the hypothalamic-pituitary-gonadal axis, resulting in a reduction of testosterone level (38, 39). Our research indicated that the testosterone level and sperm count of epididymal tail were decreased in the MC group as well, which was probably due to hyperlipidemia. However, in the CO, COS, and SN groups were significantly higher than those in the MC group at day 60. We inferred that the three groups of test substances significantly increased the testosterone levels and sperm counts in the epididymal tail of male rats with hyperlipidemia. However, only COS and SN groups significantly increased testosterone levels at day 30, suggesting that CO mixed with squalene could play a role in combining the effect of promoting the level of testosterone and the number of epididymal tail sperm, and this effect was better than that of pure CO. This finding might be because squalene can participate in cholesterol biosynthesis and various biochemical reactions in the body; accelerate the synthesis of steroid hormones, such as testosterone (40, 41); increase the activity of SOD and blood oxygen content (15); promote blood circulation; and reduce blood lipid levels, which in turn improve sexual function. At day 60, the testosterone level and sperm count in the epididymal tail did not increase compared with the day 30, suggesting that the testosterone level and sperm count in the epididymis did not increase with feeding time. Therefore, we considered that the optimal feeding duration of CO and squalene on hyperlipidemia-induced sexual dysfunction rats was 30 days.

Current animal studies showed that hyperlipidemia can impair erectile function by changing the morphological structures of sexual organs, e.g., penile corpus cavernosum lesions. Li et al. (42) assessed erectile function by performing cavernous nerve electrostimulation followed by intracavernosal pressure/mean arterial pressure measurements, as well as plasma lipid profile assessment, and then pointed out that hyperlipidemia caused cavernous fibrosis by increasing plasma lipid levels, fibrosis and apoptosis, decreasing smooth muscle/collagen ratio and autophagy. The organ coefficient and pathological results of this study showed that the organ coefficient of penis decreased significantly and that the penis and testis showed pathological changes in different degrees in the MC group, such as the number of smooth muscle cells and cavernous sinus decreased significantly in the corpus cavernosum penis, and the smooth muscle of the cavernous body were distributed unevenly and arranged disorderly and loosely, all levels of spermatogenic cells in the testis were arranged disorderly, and some spermatogenic cells showed necrosis, apoptosis, and nuclear pyknosis. These findings were consistent with those of previous reports (42–44). Compared with the MC group, the CO, COS, and SN groups had significantly increased penile organ coefficient and no pathological change in penis and testis, suggesting that the three groups of test substances could improve the damage of penis and testis and promote the recovery of erectile function.

In conclusion, results demonstrated that the addition of squalene to CO as a combining substance to feed hyperlipidemia-induced reproductive damage rats for 30 days could play an important role in combining the effect of lowering blood lipid, promoting the level of testosterone and the number of epididymal tail sperm, improving the damage of penis and testis, and promoting the recovery of erectile and sexual function. However, further studies should be carried out to elucidate the molecular mechanisms of CO and squalene in lowering blood lipid and improving sexual function *in vivo*.

## Data availability statement

The original contributions presented in this study are included in the article/supplementary material, further inquiries can be directed to the corresponding author.

## Ethics statement

This animal study was reviewed and approved by Ethics Committee of Gannan Medical University.

## Author contributions

QX: methodology, investigation, and writing—original draft preparation. ML: methodology, validation, investigation, formal analysis, and data curation. GC and QZ: investigation and resource. YG: validation, writing—review and editing, and software. JL: conceptualization, writing—review and editing, funding acquisition, and supervision. All authors contributed to the article and approved the submitted version.
